# Casticin-Induced Inhibition of Cell Growth and Survival Are Mediated through the Dual Modulation of Akt/mTOR Signaling Cascade

**DOI:** 10.3390/cancers11020254

**Published:** 2019-02-22

**Authors:** Jong Hyun Lee, Chulwon Kim, Jae-Young Um, Gautam Sethi, Kwang Seok Ahn

**Affiliations:** 1 Department of Science in Korean Medicine, Kyung Hee University, 24 Kyungheedae-ro, Dongdaemun-gu, Seoul 02447, Korea; mirue88@nate.com (J.H.L.); sunny10526@nate.com (C.K.); jyum@khu.ac.kr (J.-Y.U.); 2 Department of Pharmacology, Yong Loo Lin School of Medicine, National University of Singapore, Singapore 117600, Singapore

**Keywords:** casticin, Akt/mTOR, cancer, apoptosis

## Abstract

The Akt/mTOR signaling cascade is a critical pathway involved in various physiological and pathological conditions, including regulation of cell proliferation, survival, invasion, and angiogenesis. In the present study, we investigated the anti-neoplastic effects of casticin (CTC), identified from the plant *Vitex rotundifolia* L., alone and/or in combination with BEZ-235, a dual Akt/mTOR inhibitor in human tumor cells. We found that CTC exerted a significant dose-dependent cytotoxicity and reduced cell proliferation in a variety of human tumor cells. Also, CTC effectively blocked the phosphorylation levels of Akt (Ser473) and mTOR (Ser2448) proteins as well as induced substantial apoptosis. Additionally treatment with CTC and BEZ-235 in conjunction resulted in a greater apoptotic effect than caused by either agent alone thus implicating the anti-neoplastic effects of this novel combination. Overall, the findings suggest that CTC can interfere with Akt/mTOR signaling cascade involved in tumorigenesis and can be used together with pharmacological agents targeting Akt/mTOR pathway.

## 1. Introduction

Most people diagnosed with cancers are treated with chemotherapy, surgery, radiation therapy. They may also receive immunotherapy, targeted and hormonal therapies. Sometimes cancer can be controlled by surgery, but if the cancer has spread, the effect of surgical operations may be limited. In the case of radiation and chemotherapy, it is difficult to avoid side effects because these treatments can also affect normal rapidly growing cells [1,2]. Many novel cancer therapies are currently being developed worldwide and the goal is to achieve optimum treatment for the patients with relatively lower side effects. Natural compounds can be obtained from a variety of sources, including plants [3], animals [4], microorganisms [5], and can be employed as pharmacological agents for cancer treatment [6,7,8]. Thus, natural compounds with broad modes of action are being explored for their potential to treat various malignancies [4,9,10,11,12,13,14,15,16].

Casticin (CTC), also known as vitexicarpin or 3′,5-dihydroxy-3,4′,6,7-tetramethoxyflavone, is a member of the class of compounds known as 7-O-methylated flavonoids [17,18]. It has been found to be practically insoluble in water and a weak acidic compound [19]. CTC is one of the components of the plant *Vitex agnus-castus L.* and can be found in fruits, herbs and spices [20]. Prior studies have shown that CTC can suppress the proliferation in human myeloid leukemia cells [21,22], and induce substantial apoptosis in human gall bladder cancer cells [23], ovarian cancer cells [24], cervical cancer cells through the induction of Jun N-terminal kinase [25], as well as lung cancer cells via mitochondrial pathway. CTC can also enhance tumor necrosis factor (TNF)-related apoptosis-inducing ligand (TRAIL). apoptosis in human colon cancer cells [26]. In addition, CTC can exert anti-inflammatory effects in preclinical models [27,28], and can abrogate cellular migration in mouse melanoma cells [29,30]. Here, this study was designed to explore the anti-cancer activities of CTC on a variety of human cancer cells and investigate the potential mechanisms underlying its actions.

The Akt/mTOR is an intracellular signaling pathway that is crucial for regulating both the cell cycle and tumorigenesis. It can also mediate many aspects of cellular functions, including nutrient uptake, cell proliferation and survival [31]. It has been demonstrated that frequent overactivation of Akt/mTOR is often encountered in several types of solid tumors and in hematological malignancies [32,33,34,35,36,37,38,39]. This pathway may be activated by number of receptor tyrosine kinases, including the epidermal cell growth factor receptor (EGFR) family and insulin-like growth factor receptor (IGFRs). AKT, also known as protein kinase B (PKB), is known to be the central node of this signaling pathway, and can be phosphorylated at Thr308 by PDK1 and at Ser473 by mTOR complex 2 (mTORC2), which increases its kinase activity [40]. Activated Akt can regulate cellular processes including cell survival, proliferation and growth and act downstream of PI3K [41]. mTOR (mammalian target of rapamycin) is a major protein in this pathway that acts both upstream and downstream of AKT [42]. It is active component of multi protein complex, target of rapamycin complex TORC1 and TORC2 [33], and regulates protein synthesis necessary for cellular growth, proliferation, angiogenesis and other cellular functions [43]. Since Akt/mTOR pathway can be involved in several important processes as described above, identification of active drugs targeting this pathway can be expected to have a major impact on various therapeutic strategies against cancer.

In this work we analyzed whether CTC can exert its anticancer effects against diverse human cancer cells and the potential molecular mechanisms involved in its action. We also sought to determine whether modulation of the Akt/mTOR signaling pathway, in particular by CTC, could mediate its anti-neoplastic actions against tumor cells. Also, the combinatorial anticancer potential of CTC along with pharmacological dual phosphatidylinositol 3-kinase (PI3K)-mTOR inhibitor, BEZ-235 was systematically examined in cancer cells.

## 2. Results

### 2.1. CTC Inhibits Cellular Growth in Several Human Cancer Cells

**Figure 1:**
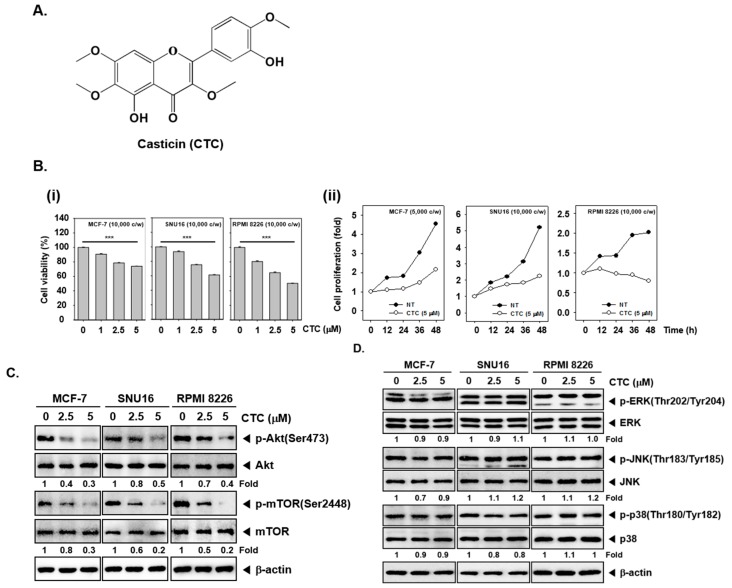
CTC inhibits cell viability and proliferation through Akt/mTOR signaling pathway in several cancer cells. (**A**) The chemical structure of casticin (CTC). (**B-i**) Effect of CTC on cell viability. Several cancer cells MCF-7, SNU16, and RPMI 8226 (1 × 10^4^ cells/well) were treated with the indicated concentrations of CTC for 24 h. Thereafter, cell viability was determined by MTT assay. (**B-ii**) Effect of CTC on cellular proliferation. MCF-7, SNU16 and RPMI 8226 cells (1 × 10^4^ cells/well) were treated with 5 µM of CTC for the indicated times. The cell proliferation was measured using the MTT assay. Abbreviation: NT = non-treated and c/w = cells per wells. (**C**) Effect of CTC on Akt signaling cascade. The cells were treated with the indicated concentrations of CTC for 9 h. Whole-cell extracts were prepared, and subjected to western blot analysis using antibodies against p-Akt(Ser473), Akt, p-mTOR(Ser2448), mTOR. (**D**) Equal amounts of lysates were analyzed by western blot analysis as described in panel C above.

To evaluate the effects of these CTC on the growth of human different cell lines, the inhibitory potential of CTC on viability was determined in human breast cancer MCF-7 cells, gastric cancer SNU16, and myeloma RPMI 8226 cells. We found that the cell viability decreased in a dose-dependent manner in cells treated with CTC. The cytotoxicity was 26% in MCF-7 cells, 39% in SNU16 cells, and 49% in RPMI8226 cells respectively, after treated with 5 μM CTC compared to non-treated group. The IC_50_ values ranging from 6 to 8.5 µM (8. 5 µM for MCF-7, 7 µM for SNU16, 6 µM for RPMI8226) (Figure 1B-i). Interestingly, the data also showed that CTC inhibited cell proliferation in in a time-dependent manner in three cancer cell lines (Figure 1B-ii).

### 2.2. CTC Suppresses Activation of Akt/mTOR Signaling Pathway

We investigated the effect of CTC on the Akt/mTOR and MAPKs signaling pathways, which are closely associated with cell proliferation and survival in tumor cell lines. Interestingly, the phosphorylation levels of Akt and mTOR were markedly decreased by CTC in MCF-7, SNU16, and RPMI 8226 cells (Figure 1C); however, phosphorylation level of members of mitogen activated protein kinases (MAPKs) signaling cascade, such as ERK, JNK, and p38 remained unchanged (Figure 1D).

### 2.3. CTC Increases Accumulation of Cells in Sub-G1 Phase and Induces Apoptosis

**Figure 2:**
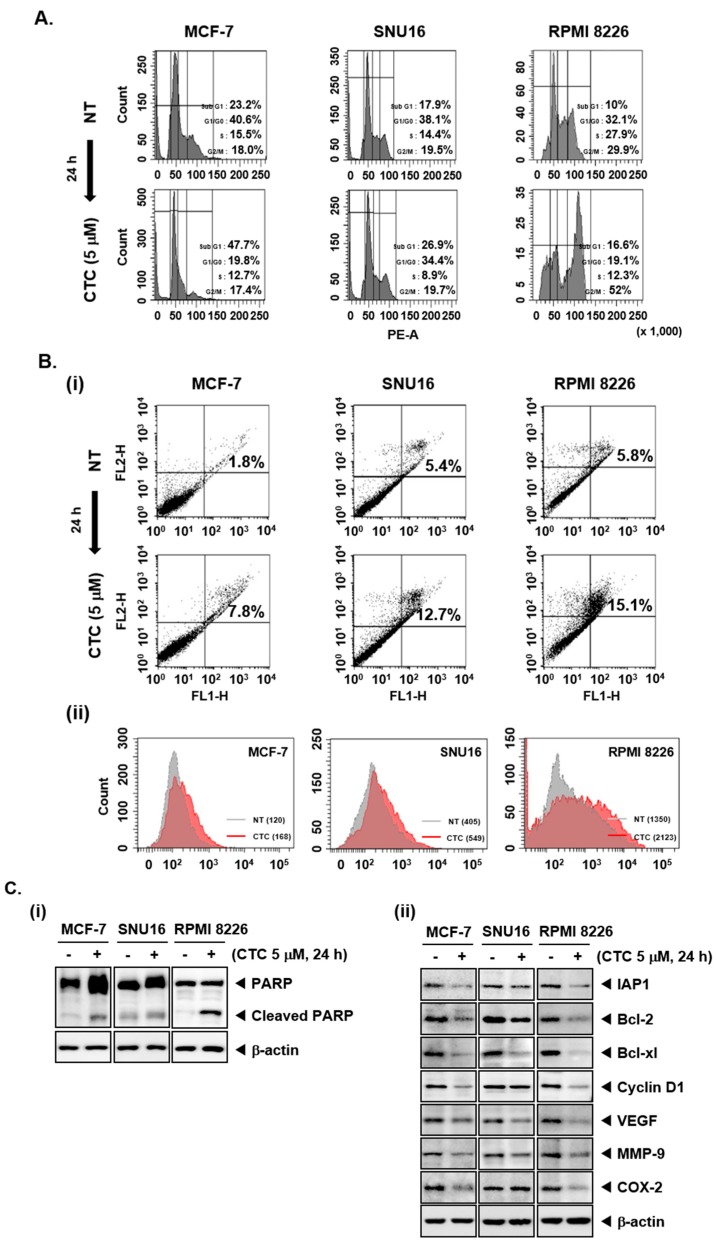
CTC induces apoptosis and reduces expression of Akt/mTOR regulated gene products. (**A**) The cells were treated with 5 µM of CTC for 24 h, harvested, stained with propidium iodide (PI), and analyzed using a flow cytometer. (**B-i**) The cells were treated with 5 µM of CTC for 24 h, harvested, stained with FITC-conjugated anti-Annexin V, and analyzed using a flow cytometer. (**B-ii**) The cells were treated with 5 µM of CTC for 24 h, harvested, fixed, incubated with a TUNEL reaction solution, and analyzed using a flow cytometer. (**C-i**,**ii**) The cells were treated with 5 µM of CTC for 24 h and western blot analysis was performed as described above in panel 1C. Abbreviation: NT = non-treated.

As the cells accumulated in the Sub-G1 phase represent the apoptotic population [44], flow cytometry was first performed to study the pro-apoptotic effect of CTC in MCF-7, SNU16, and RPMI 8226 cells. An increase in the percentage of the cells in sub-G1 phase compared with controls was noted, thereby suggesting that these cells may undergo apoptosis (Figure 2A). In addition, after treatment with 5 µM of CTC, annexin V staining was carried out by flow cytometry. As shown in Figure 2B-i, the percentage of apoptotic cells were increased compared with non-treated population in MCF-7, SNU16, and RPMI 8226 cells. Moreover, as shown in Figure 2B-ii, highly significant differences in mean values between NT and CTC treated groups for TUNEL positive cell population was noted. The mean value was found to increase from 120 to 168, 405 to 549, and 1350 to 2123 in MCF-7, SNU16, and RPMI 8226 cells, respectively. These results confirm that CTC treatment caused substantial apoptosis in tumor cell lines.

### 2.4. CTC Induces Apoptosis via the Suppression of Various Oncogenic Proteins

We also examined the effect of CTC on the proteolytic cleavage of poly (ADP-ribose) polymerase (PARP) which is regarded as an important indicator of apoptosis [45]. CTC treatment resulted in substantial PARP cleavage in MCF-7, SNU16, and RPMI 8226 cells (Figure 2C-i). Additional western blot analysis showed that CTC also markedly suppressed the expression of anti-apoptotic proteins (IAP1, Bcl-2, and Bcl-xl), the cell cycle regulator protein (Cyclin D1), angiogenic gene product (VEGF), metastatic gene product (MMP-9), and the inflammatory protein (COX-2) (Figure 2C-ii).

### 2.5. Pre-Treatment with CTC Abrogates EGF-Induced Oncogenic Signaling Cascade

**Figure 3:**
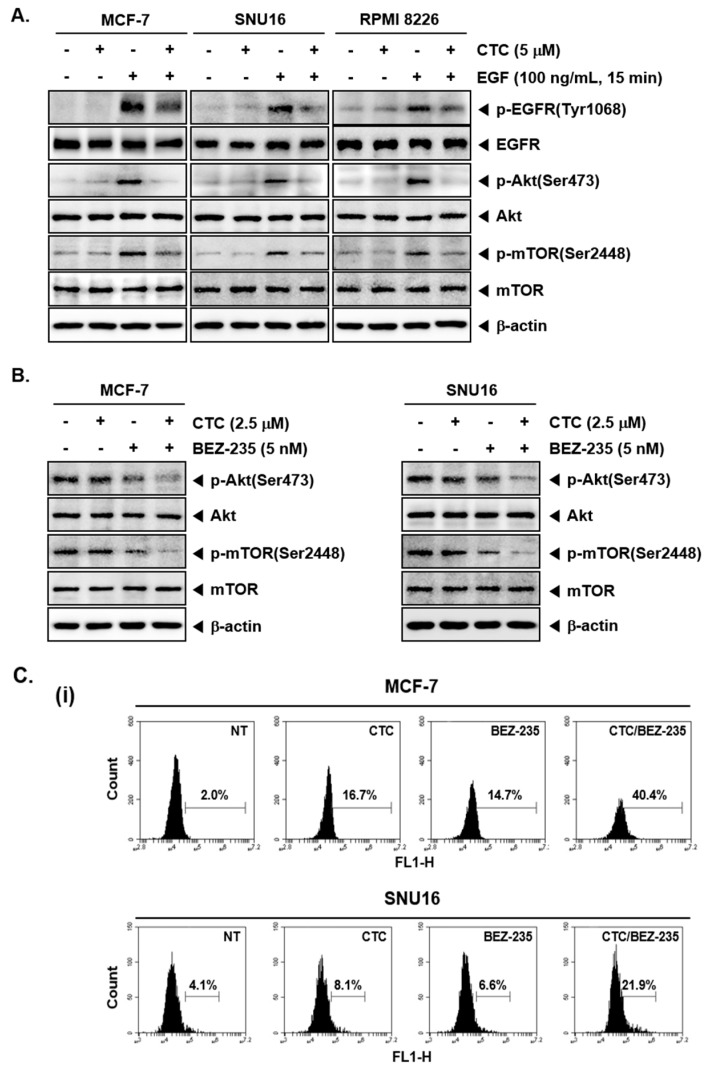

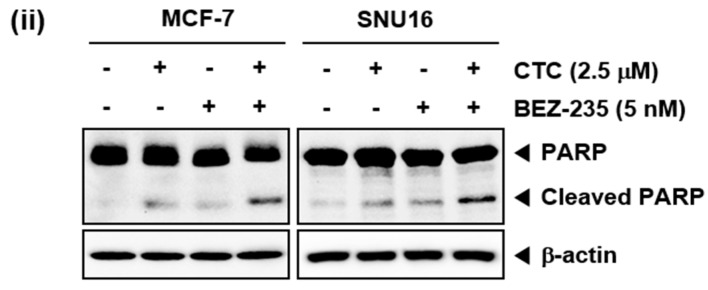
CTC suppresses EGF-stimulated Akt/mTOR signaling pathway. (**A**) MCF-7, SNU16, and RPMI 8226 cells were serum starved 12 h and treated with 5 µM of CTC for 9 h, followed by stimulation with 100 ng/mL of EGF for 15 min and western blot analysis was performed as described above in panel 1C. (**B**) The cells were treated with 2.5 µM of CTC alone or combined with 5 nM BEZ-235 for 9 h. Whole-cell extracts were prepared, and western blot analysis was performed as described above in panel 1C. (**C-i**) The cells were treated with 2.5 µM of CTC alone or in combination with 5 nM BEZ-235 for 24 h and TUNEL staining done as described in panel 2B-ii above. (**C-ii**) The cells were treated with 2.5 µM of CTC alone or combined with 5 nM BEZ-235 for 24 h and western blot analysis was performed as described above in panel 1C.

Epidermal growth factor (EGF) stimulates cell growth and differentiation by binding to its receptor, EGFR [46,47,48] in order to induce the activation of downstream Akt and mTOR signaling pathways [49]. To further investigate the effect of CTC on the EGFR signaling pathway, MCF-7 and SNU16 cells were starved for 12 h with serum free culture and then treated with CTC in the presence or absence of EGF. Interestingly, in EGF-stimulated cells, the phosphorylation levels of EGFR, Akt, and mTOR proteins were significantly increased compared with the respective control group. However, this increase was substantially attenuated upon exposure to CTC (Figure 3A).

### 2.6. CTC and BEZ-235 Efficiently Inhibits Akt/mTOR Signaling Pathway

BEZ-235 has been reported to attenuate activation of PI3K/mTOR signaling proteins and induce apoptosis as a dual PI3K-mTOR inhibitor [50]. In our study, western blot analysis revealed that BEZ-235 at sub-optimal doses slightly blocked the expression of p-Akt and p-mTOR, whereas, BEZ-235 in combination with CTC substantially inhibited the expression of p-Akt and p-mTOR in these cells (Figure 3B). 

### 2.7. CTC and BEZ-235 Can Exhibit Enhanced Apoptosis in MCF-7 and SNU16 Cells

We next determined the percentage of apoptotic cells by the well-established TUNEL assay, which enables the visualization of apoptotic cells using an in situ end-labelling technique that labels DNA breaks in apoptotic cells [51]. As shown in Figure 3C, CTC alone, at 2.5 µM, resulted in 16.7% apoptosis, while treatment with BEZ-235 alone, at 5 nM, led to 14.7% apoptosis. However the combinatorial application of both agents produced a greater effect, resulting in 40.4% apoptosis in MCF-7 cells (Figure 3C-i *Top*). In SNU16 cells, exposure to CTC alone resulted in 8.1% cell apoptosis, whereas treatment with BEZ-235 alone led to 6.6% cell apoptosis. However, exposure of cells to the combination of CTC and BEZ-235 resulted in 21.9% apoptosis (Figure 3C-i *bottom*). Furthermore, we observed that PARP cleavage were further increased by the combination treatment of two drugs together (Figure 3C-ii).

### 2.8. Knockdown of Akt by si-RNA Reverses the Growth Inhibitory and Apoptotic Effects of CTC

**Figure 4:**
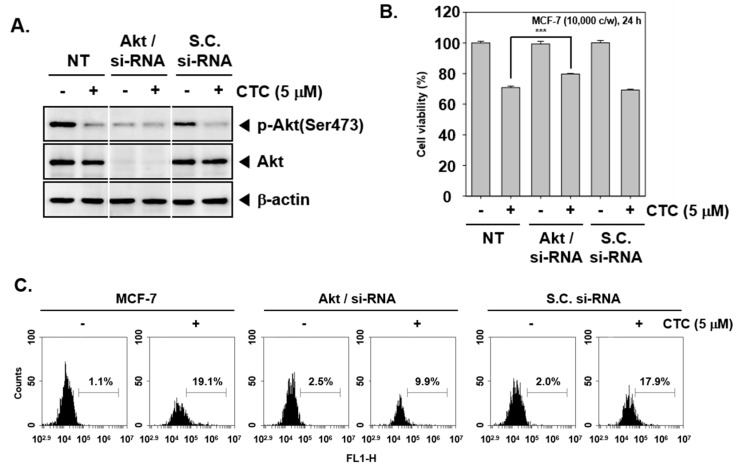
The role of Akt in growth inhibitory effects of CTC. (**A**) MCF-7 cells were transiently transfected with Akt/si-RNA or scrambled si-RNA (control). Then, whole cell extracts were prepared and western blot analysis was performed as described above in panel 1C. (**B**) Akt/scrambled si-RNA transfected MCF-7 cells were treated with 5 µM of CTC for 24 h. Thereafter, cell viability was measured using MTT assay; (**C**) Cellular apoptosis was determined using TUNEL assay.

To provide a direct evidence that apoptotic effects induced by CTC are due to inhibition of Akt pathway, transient transfections were carried out using Akt/si-RNA and scrambled si-RNA (control) in MCF-7 cells. The data demonstrated that Akt expression was substantially blocked upon transfection with Akt/si-RNA (Figure 4A). Subsequently, When Akt/si-RNA and scrambled si-RNA transfected MCF-7 cells were treated with CTC for 24 h, cell viability were performed using MTT assay. As shown in Figure 4B, MCF-7 cells transfected with scrambled si-RNA, cell viability were reduced to 69% upon treatment with CTC. In contrast, transfection of CTC-treated cells with Akt/si-RNA slightly inhibited CTC-induced reduction in cell viability as compared to the control group.

Also, as shown in Figure 4C, upon transfection with scrambled si-RNA drug treatment alone resulted in substantial apoptosis (19.1%) compared with the control group (1.1%), whereas only 9.9% of the cells in the knockdown group were found to be apoptotic.

## 3. Discussion

We report here that CTC can significantly inhibit the cell viability of three different tumor cell lines (breast, gastric, myeloma cancer cells). These findings are in agreement with prior reports where CTC was observed to suppress the proliferation of diverse tumor cells [52]. The Akt/mTOR pathway has been reported to be frequently deregulated in human cancers, regulating the apoptotic response through its ability to interact with a number of key players in the apoptotic process [37]. We found that CTC can effectively suppress the Akt activation at Ser residue 473 in a concentration-dependent manner in MCF-7, SNU16, and RPMI 8226 cells, which may contribute to its anticancer activity. A previous study has reported that CTC can also suppress self-renewal and invasion through the negative regulation of Akt signaling pathway in lung cancer cells. [53]. Our results also indicate that CTC abrogated the mTOR activation at Ser residue 2448 in tumor cells. 

Mitogen-activated protein kinases (MAPKs) such as ERK, JNK and p38 can regulate tumorigenesis and associated processes of proliferation, migration and survival. The Akt/mTOR and the MAPKs signaling pathways can be concurrently constitutively activated in several human cancers [54] and possible cross talks between these two cascades can drive tumor progression [55]. Previous reports have shown that CTC can inhibit phosphorylation of Akt, PI3K and MAPK in lung epithelial cells [56]. Thus, we also examined the ability of CTC to modulate MAPK signaling cascades but noted that this agent did not affect activation of ERK, JNK, and p38 proteins in tumor cells analyzed.

The cell cycle is a conserved mechanism by which eukaryotic cells replicate themselves. This can be divided into three stages: interphase, mitotic stage (M) phase, and cytokinesis. During interphase (G1, S, G2), cells grows, accumulating nutrients needed for mitosis, and replicate DNA. In M phase, the chromosomes are separated and during the final stage, cytokinesis, the chromosomes and cytoplasm are separated into two new daughter cells. Cells that have stopped dividing are known to enter a quiescent state called the G0 phase [57]. According to the literature, CTC can induce apoptosis through causing cell cycle arrest in oral cancer cells [58]. We also noted that CTC can induce accumulation of the cells in the sub G1 phase of cell cycle in and induce apoptosis in MCF-7, SNU16, and RPMI 8226 cells as evident by positive annexin V and TUNEL staining. Previous studies have also reported that CTC can induce early cell death in a concentration-dependent manner in bladder cancer NOZ and SGC996 cells [23]. Moreover, CTC also inhibited the expression of Akt/mTOR-controlled gene products such as anti-apoptotic (IAP2, Bcl-2, and Bcl-xl), cell cycle regulator (Cyclin D1), angiogenetic (VEGF), metastatic (MMP-9), and inflammation (COX-2). Interestingly, we also noted that the deletion of Akt by si-RNA can effectively abrogate the observed apoptotic effects of CTC, thereby indicating that downregulation of various oncogenic proteins may be caused by direct modulation of Akt activation by CTC. 

BEZ-235 is a dual PI3K-mTOR inhibitor that can target activation of PI3K and mTOR kinases and has been actively used against various cancers [59]. It is well tolerated, exhibits disease arrest upon oral administration, and improves the efficacy of other anticancer drugs when used in combinatorial setting [60]. Moreover, it has been found that BEZ-235 can synergistically potentiate the antitumor effects of cisplatin in bladder cancer cells though the cell cycle progression [61]. We noted that CTC in combination with BEZ-235 can effectively down modulate the phosphorylation of AKT/mTOR proteins and induce substantial apoptosis in tumor cells. This finding is quite intriguing as combinatorial antineoplastic effects of various flavonoids have been previously reported with different anti-cancer agents commonly used in the clinic [62,63,64,65,66]. Our group has also reported that isorhamnetin, a methylated metabolite of dietary flavonoid quercetin, can abrogate the activation of master transcription factor NF-κB [67,68,69,70,71,72] and thus significantly enhance the anti-tumoral effects of capecitabine in gastric cancer xenograft mouse model [72]. Overall, our data suggested that CTC can be potentially employed in combination therapy against malignancies, however these results have to be further validated in preclinical studies.

## 4. Materials and Methods

### 4.1. Reagents

Casticin (CTC, Figure 1A) was purchased from Biopurify Phytochemicals Ltd. (Sichuan, China). Stock solution of CTC (100 mM) was prepared in dimethyl sulfoxide, stored at −80 °C, and diluted in cell culture medium for use. Dimethyl sulfoxide (DMSO), 3-(4,5-dimethylthiazol-2-yl)-2,5-diphenyl-tetrazolium bromide (MTT), sodium dodecyl sulfate (SDS), and ribonuclease A from bovine pancreas were purchased from Sigma–Aldrich (St. Louis, MO, USA). Bovine serum albumin was purchased from Biosesang (Sungnam, Korea). RPMI1640 media, fetal bovine serum (FBS), and antibiotic-antimycotic mixture were obtained from Thermo Scientific HyClone (Waltham, MA, USA). ApoScan^TM^ Annexin V FITC apoptosis detection kit was purchased from bio-bud (Seoul, Korea). TUNEL enzyme and TUNEL label were purchased Roche (Basel, Switzerland). BEZ-235 obtained from Selleckchem (Houston, TX, USA). Acryl-bisacrylamide (29:1) was obtained from ELPIS Biotech (Daejeon, Korea).

### 4.2. Cell Lines and Culture Conditions

Human breast adenocarcinoma cell line MCF-7 cells and human myeloma cell line RPMI 8226 cells were purchased from the American Type Culture Collection (ATCC, Manassas, VA, USA). Human gastric carcinoma SNU16 cells were purchased from the Korean Cell Line Bank (Seoul, Korea). All cells were cultured in RPMI 1640 medium containing 10% FBS and 1% penicillin-streptomycin (P/S). The cells were maintained at 37 °C in a humidified atmosphere 5% CO_2_. All the cultures were routinely tested and were mycoplasma-free.

### 4.3. Cell Viability Assays

To evaluate cell viability, MCF-7, SNU16, and RPMI 8226 cells were seeded (1 × 10^5^ cells/well) in a 96-well plate, and incubated at 37 °C for 12 h. The cells were treated with various concentrations of CTC for 24 h. Thereafter, 20 µL of MTT solution (2 mg/mL in PBS) was added to each well and incubated at 37 °C in the dark. After 2 h incubation, a MTT lysis solution (20% SDS, 50% dimethylformamide) was added to each well and incubated at 37 °C for overnight, and the absorbance was then measured at 570 nm by a Varioskan LUX multimode microplate reader (Thermo Scientific). Cell viability was expressed relative to untreated control cells.

### 4.4. Western Blot Analysis

To assess the effects of Akt/mTOR inhibitors and apoptosis MCF-7, SNU16, and RPMI8226 cells were treated with 0, 2.5, and 5 µM of CTC for 9 h or 24 h. To determine the inhibitory effect of EGF-induced Akt/mTOR inhibitors, MCF-7 and SNU16 cells were treated with 100 ng/mL of EGF for 15 min after pretreatment with 5 µM of CTC for 9 h. To evaluate the combined effect of CTC and BEZ-235, MCF-7 and SNU16 cells were treated with 2.5 µM of CTC or 5 nM of BEZ-235 for 9 h. Whole-cell extracts were lysed in a lysis buffer (20 mM Tris (pH 7.4), 250 mM NaCl, 2 mM EDTA (pH 8.0), 0.1% Triton X-100, 0.01 mg/mL aprotinin, 0.005 mg/mL leupeptin, 0.4 mM phenyl methane sulfonyl fluoride (PMSF), and 4 mM NaVO4) for 1 h. The lysates were then spun at 14,000 rpm for 20 min to remove insoluble material, and supernatant was transferred to a fresh tube kept on ice. Protein concentrations was performed using a Bradford protein assay [73]. Bovine serum albumin (BSA) was used as a protein standard. Equal amounts of protein (10 µg) were separated by 8–12% SDS-PAGE and electro-transferred onto nitrocellulose membrane and western blot analysis was carried out as described before [74]. Antibodies against p-Akt(Ser473), Akt, p-mTOR(Ser2448), mTOR, p-ERK (Thr202/Tyr204), ERK, p-JNK(Thr183/Tyr185), JNK, p-p38(Thr180/Tyr182), p38, PARP, Caspase-3, IAP1, Bcl-2, Bcl-xl, Cyclin D1, VEGF, MMP-9, COX-2, p-EGFR(Tyr1068), EGFR, and β-actin were used for western blots. Repeated experiments were performed twice to obtain quantitative data. Quantification of band intensities for each represented blot was performed using Image J software (National Institutes of Health (NIH), Bethesda, MD, USA).

### 4.5. Cell Cycle Analysis

To determine apoptosis, cell cycle analysis was performed using propidium iodide (PI) staining. Briefly the MCF-7, SNU16, and RPMI8226 cells were treated with 5 µM of CTC for 24 h, and then the cells were harvested, washed with cold PBS. Cell pellets were fixed with 70% cold ethanol overnight at 4 °C. The fixed cells were resuspended in 1× PBS containing 1 mg/mL RNase A, incubated for 1 h at 37 °C incubation. Cells were then washed, resuspended, and stained in PBS containing 25 µg/mL of PI for 30 min at room temperature in the dark. Stained samples were analyzed by BD Accuri C6 plus flow cytometer (BD Biosciences, San Diego, CA, USA). Acquisition and analysis of the data were performed using BD Accuri C6 plus software (version 1.0.23.1).

### 4.6. Annexin V and TUNEL Assays

The MCF-7, SNU16, and RPMI 8226 cells were treated with 5 µM of CTC for 24 h. Apoptosis was evaluated by annexin V-FITC and propidium iodide (PI) stained cells using a FITC annexin V Apoptosis Detection Kit I according to the manufacturer’s protocols. Briefly, the cells were harvested using 1% trypsin in PBS. The cell pellet was resuspended in 1× binding buffer add 5 µL of FITC Annexin V and 5 µL of PI for 15 min at room temperature in the dark. Stained samples were analyzed by BD Accuri C6 plus flow cytometer (BD Biosciences). Acquisition and analysis of the data were performed using BD Accuri C6 plus software (version 1.0.23.1).

### 4.7. Drug Combination Analyses

MCF-7 and SNU16 cells were drug-treated for 24 h with CTC, BEZ-235 or their combination; cytotoxicity was measured by MTT assay. Synergy or antagonism were determined with computer software CalcuSyn for windows (Biosoft, Cambridge, UK). In this system, synergism, additivity, or antagonism is defined by the combination index; a CI value <1 indicates the synergistic effect, a CI value of 1 indicates an additive effect and a CI value >1 indicates an antagonistic effect.

### 4.8. siRNA Transfection

siRNA transfection was performed as described before [74].

### 4.9. Statistical Analysis

Data are expressed as the mean ± S.D. In all figures, vertical error bars denote the S.D. The significance of differences between groups was evaluated by Student’s *t*-test and one way analysis of variance, (ANOVA) test. The *p* value of less than 0.05 was considered statistically significant.

## 5. Conclusions

CTC inhibited the survival and proliferation of diverse cancer cells as well as down-regulated Akt/mTOR signaling pathway and suppressed various proteins involved in anti-apoptosis, metastasis, and angiogenesis. In addition, the combinatorial treatment of CTC and BEZ-235 exhibited a significant apoptotic effects against neoplastic cells. Overall, our results conclusively demonstrate that CTC can function as a potential inhibitor of tumor cell survival and proliferation by negatively regulating Akt/mTOR activation.

## Abbreviations

MTT, 3-(4,5-Dimethylthiazol-2-yl)-2,5-diphenyltetrazolium bromide; ICC, Immunocytochemistry; RTCA, real-time cell analysis; SDS-PAGE, sodium dodecyl-polyacrylamide gel electrophoresis; HRP, horseradish peroxidase; ECL, enhanced chemiluminescence.
